# Least absolute shrinkage and selection operator type methods for the identification of serum biomarkers of overweight and obesity: simulation and application

**DOI:** 10.1186/s12874-016-0254-8

**Published:** 2016-11-14

**Authors:** Monica M. Vasquez, Chengcheng Hu, Denise J. Roe, Zhao Chen, Marilyn Halonen, Stefano Guerra

**Affiliations:** 1 Mel and Enid Zuckerman College of Public Health, The University of Arizona, 1295 North Martin Avenue, P.O. Box 245211, Tucson, AZ 85724 USA; 2 Asthma and Airway Disease Research Center, The University of Arizona, 1501 North Campbell Avenue, P.O. Box 245030, Tucson, AZ 85724 USA; 3 ISGlobal CREAL Centre, University Pompeu Fabra, Barcelona, Spain

**Keywords:** LASSO, Biomarkers, High-Dimensional, Obesity, Overweight

## Abstract

**Background:**

The study of circulating biomarkers and their association with disease outcomes has become progressively complex due to advances in the measurement of these biomarkers through multiplex technologies. The Least Absolute Shrinkage and Selection Operator (LASSO) is a data analysis method that may be utilized for biomarker selection in these high dimensional data. However, it is unclear which LASSO-type method is preferable when considering data scenarios that may be present in serum biomarker research, such as high correlation between biomarkers, weak associations with the outcome, and sparse number of true signals. The goal of this study was to compare the LASSO to five LASSO-type methods given these scenarios.

**Methods:**

A simulation study was performed to compare the LASSO, Adaptive LASSO, Elastic Net, Iterated LASSO, Bootstrap-Enhanced LASSO, and Weighted Fusion for the binary logistic regression model. The simulation study was designed to reflect the data structure of the population-based Tucson Epidemiological Study of Airway Obstructive Disease (TESAOD), specifically the sample size (*N* = 1000 for total population, 500 for sub-analyses), correlation of biomarkers (0.20, 0.50, 0.80), prevalence of overweight (40%) and obese (12%) outcomes, and the association of outcomes with standardized serum biomarker concentrations (log-odds ratio = 0.05–1.75). Each LASSO-type method was then applied to the TESAOD data of 306 overweight, 66 obese, and 463 normal-weight subjects with a panel of 86 serum biomarkers.

**Results:**

Based on the simulation study, no method had an overall superior performance. The Weighted Fusion correctly identified more true signals, but incorrectly included more noise variables. The LASSO and Elastic Net correctly identified many true signals and excluded more noise variables. In the application study, biomarkers of overweight and obesity selected by all methods were Adiponectin, Apolipoprotein H, Calcitonin, CD14, Complement 3, C-reactive protein, Ferritin, Growth Hormone, Immunoglobulin M, Interleukin-18, Leptin, Monocyte Chemotactic Protein-1, Myoglobin, Sex Hormone Binding Globulin, Surfactant Protein D, and YKL-40.

**Conclusions:**

For the data scenarios examined, choice of optimal LASSO-type method was data structure dependent and should be guided by the research objective. The LASSO-type methods identified biomarkers that have known associations with obesity and obesity related conditions.

## Background

The study of circulating biomarkers and their association with disease outcomes has become progressively complex due to advances in biotechnologies available for the measurement of these biomarkers, including multiplex technologies. Although the availability of numerous biomarkers to study disease outcomes is highly promising, high dimensional biomarker data present statistical challenges. The Least Absolute Shrinkage and Selection Operator (LASSO) [1] is a popular high dimensional data analysis method that may be utilized for these biomarker data because it can simultaneously perform regularization and variable selection, which can improve both prediction accuracy and interpretation. This method, originally proposed for the linear regression model, minimizes the residual sum of squares, subject to the sum of the absolute value of the coefficients being less than a tuning parameter [1]. For the binary logistic regression model, the residual sum of squares is replaced by the negative log-likelihood [2]. If this tuning parameter is large, there is no effect on the estimated regression parameters. However, as the tuning parameter gets smaller, this may cause some coefficients to be shrunk towards zero or set to be zero. Still, there has been extensive and ongoing research towards the improvement of this method in obtaining a more sparse and consistent solution. Therefore, the key aim of this study was to evaluate five extensions that have been proposed to improve the sparsity and consistency of the original LASSO method. A simulation study was performed to compare variable selection properties of the original LASSO method with the Adaptive LASSO (AL), Elastic Net (EN), Iterated LASSO (IL), Bootstrap-Enhanced LASSO (BL), and Weighted Fusion (WF) for the binary logistic regression model.

### LASSO

Details on penalized binomial logistic regression have been previously described [2]. Let **x**_i_ = (x_i1_, …, x_ip_)^⊺^ for i =1,…,N denote p-predictors for N observations. Assume that responses for the binary logistic regression model can take values G = 1,2. Then,

$$ \Pr \left(\mathrm{G}=1\Big|\mathrm{x}\right)=\frac{1}{1+{\mathrm{e}}^{- \left({\upbeta}_0+{{\mathbf{x}}_{\mathrm{i}}}^{\intercal}\boldsymbol{\upbeta} \right)}},\kern0.5em  \Pr \left(\mathrm{G}=2\Big|\mathrm{x}\right)=\frac{1}{1+{\mathrm{e}}^{\left({\upbeta}_0+{{\mathbf{x}}_{\mathrm{i}}}^{\intercal}\boldsymbol{\upbeta} \right)}}, $$

where β_0_ is the intercept and **β** = (β_1_, β_2_, …, β_p_)^⊺^ is a p-vector of regression parameters. This implies

$$ \log \frac{ \Pr \left(\mathrm{G}=1\Big|\mathrm{x}\right)}{ \Pr \left(\mathrm{G}=2\Big|\mathrm{x}\right)}={\upbeta}_0+{{\mathbf{x}}_{\mathrm{i}}}^{\intercal}\boldsymbol{\upbeta} . $$

The LASSO method then finds parameter values to minimize

$$ \hbox{--} \left[\frac{1}{\mathrm{N}}{\displaystyle \sum_{\mathrm{i}=1}^{\mathrm{N}}}{\mathrm{y}}_{\mathrm{i}}\cdot \left({\upbeta}_0+{{\mathbf{x}}_{\mathrm{i}}}^{\intercal}\boldsymbol{\upbeta} \right)- \log \left(1+{\mathrm{e}}^{\left({\upbeta}_0+{{\mathbf{x}}_{\mathrm{i}}}^{\intercal}\boldsymbol{\upbeta} \right)}\right)\right]+\uplambda \cdot {\displaystyle \sum_{\mathrm{j}=1}^{\mathrm{p}}}\left|{\upbeta}_{\mathrm{j}}\right| $$

where

$$ \uplambda \cdot {\displaystyle \sum_{\mathrm{j}=1}^{\mathrm{p}}}\left|{\upbeta}_{\mathrm{j}}\right| $$

is the penalty function for the LASSO.

### Adaptive LASSO

The LASSO method has shown to not always provide consistent variable selection. The LASSO penalizes all coefficients equally, even when the coefficients are large. In contrast, the AL uses adaptive weights to penalize coefficients differently [3]. AL uses a weighted penalty,

$$ \uplambda \cdot {\displaystyle \sum_{\mathrm{j}=1}^{\mathrm{p}}}{\mathrm{w}}_{\mathrm{j}}\cdot \left|{\upbeta}_{\mathrm{j}}\right|, $$

where ${w}_j=\frac{1}{{\left|{\widehat{\upbeta}}_{\mathrm{j}}\right|}^{\mathrm{v}}},\ \left|{\widehat{\upbeta}}_{\mathrm{j}}\right|$ is the maximum likelihood estimate and v > 0. The weighted penalty will allow variables with larger coefficients to receive smaller penalties and thus might provide a more consistent solution.

### Elastic net

Zou and Hastie proposed the EN to address three issues related to the LASSO [4]. The first two issues relate to highly correlated variables in the n > p situation. For highly correlated variables, the LASSO tends to choose one variable and not the others. Also, predictive performance for ridge regression was empirically observed to be better than LASSO [4]. The third issue relates to the p > n situation in which the LASSO can at most select n variables. The penalty term for EN incorporates both the ridge penalty [5] λ ⋅ ∑_j = 1_^p^β_j_^2^ and LASSO penalty λ ⋅ ∑_j = 1_^p^|β_j_|:

$$ \uplambda \cdot {\displaystyle \sum_{\mathrm{j}=1}^{\mathrm{p}}}\ \left[\frac{1}{2}\cdot \left(1-\upalpha \right)\cdot {\upbeta_{\mathrm{j}}}^2+\upalpha \cdot \left|{\upbeta}_{\mathrm{j}}\right|\right], $$

where α is a value between 0 and 1. The EN penalty is equal to the LASSO penalty when α = 1 and to the ridge penalty when α = 0. The EN selects groups of correlated variables together, shares nice properties of both the LASSO and ridge regression, and can be considered for situations with p > n.

### Iterated LASSO

The purpose of the IL is to consider the AL where the weights are based on LASSO estimates of the coefficients rather than maximum likelihood estimates [6]. An initial estimator is obtained by the IL to reduce the dimension of the model. The IL uses the LASSO to obtain an initial estimator and reduce the dimension of the model. The LASSO estimates are then used for the weighted penalty.

### Bootstrap-enhanced LASSO

The bootstrap sample x* = (x_1_*, *x*_2_*, …, x_n_*) is obtained by randomly sampling the initial data points x_1_, *x*_2_, …, x_n_ with replacement. The BL takes several bootstrapped replications of a sample and then considers the intersection of these estimates [7]. The motivation behind the BL is that if there existed several data sets from the same distribution, relevant variables would appear in all data sets and by running the LASSO for several bootstrapped replications would lead to a consistent model. As the BL may be too strict in intersecting models, it has been recommended to use a softened version of the BL (BL-S) [8]. In particular, a BL-S that considered variables that appeared in at least 90% of the bootstrap samples was shown to have better performance than the BL [8]. Similarly, for this study rather than considering a strict intersection, we a priori considered variables that appeared in at least 75% of the bootstrap samples (BL-75), consistent with other application studies [9, 10].

### Weighted fusion

The motivation behind WF is to improve the EN by utilizing additional information from the correlation structure. In particular, the WF utilizes information from correlated variables by using correlation driven weights to penalize for the pairwise differences of these coefficients [11]. The penalty term is defined as

$$ {\uplambda}_1\cdot {\displaystyle \sum_{\mathrm{j}=1}^{\mathrm{p}}\left|{\upbeta}_{\mathrm{j}}\right|}+{\uplambda}_2\cdot \mathrm{J}\left(\upbeta \right), $$

where tuning parameters λ_1_ ≥ 0, λ_2_ ≥ 0, and J is a correlation driven penalty function,

$$ \mathrm{J}\left(\upbeta \right)=\frac{1}{\mathrm{p}}\cdot {\displaystyle \sum_{\mathrm{i}<j}{\mathrm{w}}_{\mathrm{i}\mathrm{j}}\cdot {\left({\upbeta}_{\mathrm{i}}-{\mathrm{s}}_{\mathrm{i}\mathrm{j}}{\upbeta}_{\mathrm{j}}\right)}^2} $$

where ${\mathrm{w}}_{\mathrm{ij}}=\frac{{\left|{\rho}_{ij}\right|}^{\gamma }}{1-\left|{\rho}_{ij}\right|}$ are the nonnegative weights non-decreasing in |ρ_ij_|, ρ_ij_ = **x**_i_^⊺^ **x**_j_ is the sample correlation between predictors, γ > 0 is a tuning parameter, and s_ij_ is the sign of ρ_ij_. The correlation driven weights encourage correlated variables to be considered together.

### Current study

The simulation study utilized the data structure of the population-based Tucson Epidemiological Study of Airway Obstructive Disease (TESAOD). This real dataset presents data scenarios that are likely to be encountered in serum biomarker research. Examples of such scenarios may include high correlation between biomarkers, weak to moderate associations with the outcome, and sparse number of true signals. The current study seeks to compare the LASSO and LASSO-type methods for scenarios similar to the TESAOD data with the intent that findings may be useful for similar biomarker studies. In the application study, a comparison of methods including the application of each LASSO and LASSO-type method to the TESAOD data was performed for the identification of serum biomarkers of overweight and obesity.

## Methods

### Simulation study

A simulation study was performed to compare the performance of the LASSO, AL, EN, IL, BL-75, and WF methods for the binary logistic regression model. A total of five scenarios were considered for the overweight and obese outcomes as shown in Table 1. Biomarkers for the simulation study were generated from the multivariate normal distribution using the R package ‘mnormt’ (version 1.5-3) [12] and the outcomes were generated from the binomial distribution using R package ‘stats’ (version 3.1.0) [13]. Tuning parameters were estimated by using 10-fold cross validation with deviance loss. For EN, the alpha parameters were also chosen by cross validation using the sequence alpha = 0 through alpha = 1 with step size 0.1. For WF, the gamma parameter was chosen from the sequence: 0.5, 1, 2.5, 5, and 25 [11].

**Table 1:** Scenarios applied to each LASSO method

Scenario	# Non-zero coefficients	Correlation among predictors	Non-zero coefficients in binary logistic regression models	
			Overweight	Obese
1	5	0.2	0.05, -0.05, 0.1, 0.15, 0.2	0.2, 0.4, 0.6, -0.6, 0.8
2	5	0.8	0.05, -0.05, 0.1, 0.15, 0.2	0.2, 0.4, 0.6, -0.6, 0.8
3	5	0.2	0.2, 0.4, 0.6, -0.6, 0.8	1, -1, 1.25, 1.5, 1.75
4	5	0.8	0.2, 0.4, 0.6, -0.6, 0.8	1, -1, 1.25, 1.5, 1.75
5	20	0.5	0.01,0.2 from uniform distribution	0.1,0.99 from uniform distribution

The data structure of TESAOD was utilized in the simulation study design. A total of 879 subjects had 86 measured serum biomarkers, thus the choice of *N* = 1000 with 100 biomarkers was made. Simulations for *N* = 500 were also completed as in the case of stratified analyses or a smaller study population. The first four scenarios considered five biomarkers with non-zero association with the outcome (or true signals) out of the 100 biomarkers, while the fifth scenario considered 20 biomarkers truly associated with the outcome. Correlation of serum biomarkers ranged from <0.01 to 0.70 for TESAOD, thus the correlation levels of 0.20, 0.50, and 0.80 were chosen. In the TESAOD dataset, 40% of subjects were overweight and 12% were obese. Thus in the simulation study the proportion of overweight was set at 40% and that of obesity at 12%. For each scenario a total of 1000 simulated datasets were generated. Overall, five scenarios, two outcomes, and two sample sizes were considered for a total of 20 simulation studies.

To evaluate the variable selection properties of each method, we first assessed if the solution provided by each method was sparse. Second, we evaluated how well each method identified the predictors with correct non-zero and zero coefficients. In addition, we also evaluated the area under the Receiver Operating Characteristic curve (AUC) on an independent validation data set to evaluate the discriminatory predictive performance of each method. The independent validation data sets were generated for each of the 1000 simulations and for each scenario with similar data structure as the training data set. Depending on the scenario, the validation data sets had sample size of either 1000 or 500 per validation data set.

### Application study

#### Study population

TESAOD is a population-based prospective cohort study initiated in 1972 in Tucson, Arizona [14]. At study initiation, 3805 participants from a stratified cluster of 1655 white Tucson households between the ages of 6 to 95 were enrolled. At study baseline, participants completed a standardized questionnaire, had height and weight measured by a study nurse, and had a sample of their blood collected. For the current study, serum biomarkers were measured on 879 non-Hispanic white subjects who were between the ages of 21 to 70 years at the 1972 baseline survey and for whom a baseline serum sample with sufficient volume was available. The University of Arizona Institutional Review Board approved the TESAOD study.

#### Biomarkers

Cryopreserved serum samples were analyzed at the Myriad-Rules Based Medicine (RBM) facilities (Austin, TX) using the Human Multi-Analyte Profile panel version 1.6, a bead based suspension multiplex assay based on Luminex immunoassay technology [15]. A total of 87 serum biomarkers were measured for 879 subjects (Table 8 in the Appendix). Additionally, five serum biomarkers were analyzed locally at the Arizona Respiratory Center (ARC) laboratory, namely Soluble CD14 (R&D Systems Inc., Minneapolis, MN), Club Cell Secretory Protein (CC16) (BioVendor, Asheville, NC), Surfactant Protein D (SPD) (Hycult Biotech Inc., Plymouth Meeting, PA), and YKL-40 (Quantikine Human CHI3L1 immunoassay by R&D, Inc., Minneapolis, MN, USA, and Abingdon, UK) using commercially available enzyme-linked immunosorbent assays and C-Reactive Protein (CRP) (Immulite 2000, Siemens Diagnostics, Tarrytown, NY) using the commercially available enzymatic solid-phase chemiluminescent immunometric assay.

A total of 86 biomarkers were considered for the application study, of which 81 were measured by Myriad-RBM and five were measured locally at the ARC. Of the 87 biomarkers measured by Myriad-RBM, six were not considered. Factor VII, Insulin, and Prostate Specific Antigen, Free were dropped as we only had measurements for 322 of the 879 subjects. Biomarkers that had greater than 90% undetectable values (i.e., Interleukin-2, Interleukin-12p70, and Lymphotactin) were also not considered. Missing data were present for Alpha-2 Macroglobulin (3 subjects), CC16 (2 subjects), YKL-40 (4 subjects), Eotaxin (1 subject), and SPD (3 subjects). Missing values were replaced with the median value for the given biomarker. Biomarker measurements fell into one of the following categories: undetectable values, normal values, and high values (Table 10 in the Appendix). Biomarkers that had less than 15% of undetectable values were considered as continuous. These biomarkers were evaluated for normality and were log transformed to obtain approximate normality when appropriate. Their concentrations were analyzed in statistical analyses as standardized values. Samples used to measure the biomarker concentrations were run using different batches in the laboratory. Variability in measurement may be introduced by not running samples in one batch. To adjust for any batch effects, the ComBat function as part of the R package ‘sva’ (version 3.10.0) was used [16, 17]. Biomarkers that had between 15 and 50% undetectable values were categorized at their median value. Biomarkers that had between 50 and 90% undetectable values were categorized at their detection limit.

#### Statistical analysis

For the TESAOD study data with the panel of 86 biomarkers, each LASSO and LASSO-type method was applied to two separate binary logistic regression models, one comparing the 306 overweight subjects with the 463 normal-weight subjects, and the other comparing the 66 obese subjects with the 463 normal-weight subjects. Furthermore, we additionally applied each LASSO-type method to a binary logistic regression model comparing the 372 overweight or obese subjects to the 463 normal weight subjects. In addition, separate univariable binary logistic regression models were performed to verify that each biomarker was independently associated with each outcome. For the BL-75, we report the median values for the variables that appeared in at least 75% of the bootstrap samples.

The analyses of simulated and real data were performed using R versions 2.15.1 and 3.0.2 and Stata version 12.0 (Statacorp LP, College Station, TX, USA). The R packages glmnet (version 1.9-8) [2, 18], lqa (version 1.0-3) [19], and ROCR (version 1.0-5) [20, 21] were used.

## Results

### Simulation results

Table 2 shows sparsity of results for all methods in each of the five scenarios. The only method that did not provide the most sparse solution for either the overweight or obese outcome across all scenarios was the EN. When considering scenarios with weak associations of biomarkers with the outcome (scenarios 1 and 2) and scenarios with highly correlated variables (scenarios 2 and 4), the IL provided the most sparse solution for the overweight outcome and for the obese outcome, the LASSO, IL, BL-75, and WF methods provided the most sparse solution given a particular scenario. For scenario 5 that considers a moderate number of true signals with moderate correlation, the IL provided the most sparse solution for the overweight outcome and the BL-75 provided the most sparse solution for the obese outcome.

**Table 2:** Median (minimum, maximum) number of selected non-zero coefficients from 1000 simulated data sets

	*N* = 1000					*N* = 500				
Scenario	1	2	3	4	5	1	2	3	4	5
True number	5	5	5	5	20	5	5	5	5	20
Overweight										
LASSO	7 (0,38)	5.5 (1,41)	19 (4,61)	9 (2,56)	19 (9,56)	4 (0,37)	5 (0,43)	18 (4,50)	8 (2,49)	16 (5,47)
Adaptive LASSO	16 (1,45)	14 (1,48)	18 (4,49)	19 (2,51)	21 (4,52)	16 (0,46)	12 (1,43)	18 (3,45)	18 (1,45)	19.5 (3,45)
Elastic Net	12 (0,100)	10 (1,100)	23 (4,62)	13 (2,65)	28 (10,100)	12 (0,100)	12 (0,100)	22 (4,84)	11 (2,62)	27 (7,100)
Iterated LASSO	7 (0,41)	5 (1,34)	18 (4,46)	9 (2,43)	16 (5,41)	4 (0,32)	4 (0,35)	16 (2,38)	7 (1,39)	13 (3,37)
Bootstrap-Enhanced LASSO-75	12 (1,28)	10 (1,26)	18 (7,38)	17 (4,40)	20 (10,40)	10 (0,29)	9 (0,28)	16 (5,37)	13 (3,34)	16 (5,35)
Weighted Fusion	27 (0,99)	27 (1,100)	23 (10,76)	21 (10,74)	34 (18,100)	13 (0,100)	13 (0,100)	11 (5,66)	9 (4,100)	56 (9,100)
Obese										
LASSO	17 (4,51)	7 (2,59)	26 (8,56)	16 (3,58)	37 (20,59)	15 (2,45)	7 (1,40)	22.5 (5,54)	14 (3,50)	28 (14,52)
Adaptive LASSO	17 (3,50)	17 (1,47)	18 (5,57)	19 (3,47)	35 (14,57)	19 (1,51)	15 (1,48)	19 (4,48)	17 (2,48)	28 (6,48)
Elastic Net	21 (5,74)	11 (2,75)	29 (6,76)	19 (4,77)	47 (22,100)	20 (2,100)	11 (1,100)	26 (5,74)	19 (3,87)	42 (14,100)
Iterated LASSO	16 (4,40)	7 (1,44)	20 (5,40)	13 (3,38)	24 (12,40)	14 (1,39)	6 (1,34)	16 (4,36)	11 (2,40)	17 (7,34)
Bootstrap-Enhanced LASSO-75	17 (5,37)	14 (2,34)	20 (7,39)	16 (5,34)	22 (12,34)	14.5 (5,36)	11 (1,27)	13 (5,24)	10 (3,20)	16 (8,25)
Weighted Fusion	12 (5,68)	10 (2,99)	43 (5,61)	36 (3,53)	39 (22,99)	38 (1,100)	4 (1,100)	26 (12,87)	22 (4,70)	47 (14,100)

Tables 3 and 4 show each method’s ability to correctly identify the non-zero and zero coefficients for each of the five scenarios. Considering *N* = 1000 with 100 biomarkers (Table 3), for the overweight outcome, the WF outperformed the other methods in identifying the non-zero coefficients, but performed the worst in identifying the zero-coefficients. The IL identified more of the zero-coefficients correctly. For the obese outcome, one method did not clearly outperform the other methods. Results for simulations with *N* = 500 with 100 biomarkers are shown in Table 4. For the overweight outcome, the WF tended to choose more of the non-zero coefficients correctly and the IL tended to identify more of the zero-coefficients correctly. For the obese outcome, the WF outperformed the others at identifying the correct non-zero coefficients, but tended to incorrectly include more noise variables.

**Table 3:** Median (minimum, maximum) number of correctly identified non-zero and zero coefficients, *N* = 1000, 1000 simulated datasets

Scenario	1		2		3		4		5	
Type	Non-Zero	Zero	Non-Zero	Zero	Non-Zero	Zero	Non-Zero	Zero	Non-Zero	Zero
Truth	5	95	5	95	5	95	5	95	20	80
Overweight										
LASSO	3 (0,5)	91 (61,95)	3 (1,5)	92 (56,95)	5 (4,5)	81 (39,95)	4 (2,5)	89 (43,95)	13 (8,18)	74.5 (39,80)
Adaptive LASSO	3 (0,5)	82 (54,95)	2 (1,5)	83 (50,95)	5 (4,5)	82 (51,95)	4 (2,5)	81 (48,95)	9 (4,15)	68 (41,80)
Elastic Net	3 (0,5)	86 (0,95)	3 (1,5)	88 (0,95)	5 (4,5)	77 (38,95)	4 (2,5)	86 (34,95)	16 (9,20)	67 (0,80)
Iterated LASSO	2 (0,5)	91 (57,95)	2 (1,5)	93 (64,95)	5 (4,5)	82 (54,95)	3 (2,5)	90 (57,95)	11 (5,16)	76 (50,80)
Bootstrap-Enhanced LASSO-75	3 (0,5)	86 (70,95)	2 (0,4)	86 (72,95)	5 (4,5)	82 (62,93)	4 (2,5)	81 (60,94)	12 (7,16)	72 (54,80)
Weighted Fusion	4 (0,5)	72 (1,95)	5 (1,5)	72 (0,95)	5 (4,5)	77 (24,90)	4 (2,5)	77 (26,89)	20 (9,20)	65 (0,74)
Obese										
LASSO	5 (3,5)	82 (49,95)	3 (2,5)	91 (41,95)	5 (5,5)	74 (44,92)	4 (3,5)	83 (42,95)	19 (14,20)	61 (37,78)
Adaptive LASSO	5 (3,5)	82.5 (50,95)	4 (1,5)	82 (53,95)	5 (5,5)	82 (43,95)	5 (3,5)	81 (53,95)	15 (10,20)	60 (38,79)
Elastic Net	5 (4,5)	79 (26,94)	4 (2,5)	88 (25,95)	5 (5,5)	71 (24,94)	4 (3,5)	80 (23,95)	19 (15,20)	52 (0,75)
Iterated LASSO	5 (3,5)	84 (60,95)	3 (1,5)	91 (56,95)	5 (5,5)	80 (60,95)	4 (3,5)	86 (62,95)	16 (12,20)	72 (56,80)
Bootstrap-Enhanced LASSO-75	5 (3,5)	83 (63,95)	3 (1,5)	84 (64,95)	5 (5,5)	80 (61,93)	4 (2,5)	83 (66,94)	16 (10,20)	73 (62,80)
Weighted Fusion	5 (3,5)	88 (32,94)	3 (2,5)	89 (1,95)	5 (5,5)	57 (39,95)	4 (3,5)	64 (46,95)	20 (14,20)	61 (1,76)

**Table 4:** Median (minimum, maximum) number of correctly identified non-zero and zero coefficients, *N* = 500, 1000 simulated datasets

Scenario	1		2		3		4		5	
Type	Non-Zero	Zero	Non-Zero	Zero	Non-Zero	Zero	Non-Zero	Zero	Non-Zero	Zero
Truth	5	95	5	95	5	95	5	95	20	80
Overweight										
LASSO	1 (0,5)	93 (59,95)	2 (0,5)	92 (54,95)	5 (2,5)	82 (50,95)	3 (2,5)	90 (50,95)	11 (5,16)	75 (46,80)
Adaptive LASSO	2 (0,5)	81 (51,95)	2 (0,4)	84 (55,95)	5 (3,5)	81 (55,95)	4 (1,5)	81 (54,95)	7 (3,14)	68 (45,80)
Elastic Net	2 (0,5)	85 (0,95)	3 (0,5)	86 (0,95)	5 (3,5)	78 (16,95)	4 (2,5)	87 (36,95)	14 (6,20)	66.5 (0,80)
Iterated LASSO	1 (0,5)	93 (66,95)	2 (0,5)	92 (62,95)	5 (2,5)	84 (62,95)	3 (1,5)	91 (60,95)	8 (3,14)	76 (52,80)
Bootstrap-Enhanced LASSO-75	2 (0,5)	87 (69,95)	1 (0,3)	87 (68,95)	5 (3,5)	84 (62,94)	3 (1,5)	85 (65,95)	8 (4,13)	72 (54,80)
Weighted Fusion	3 (0,5)	85 (0,95)	5 (0,5)	85 (0,95)	5 (3,5)	89 (34,94)	3 (2,5)	89 (0,94)	20 (6,20)	43 (0,80)
Obese										
LASSO	4 (1,5)	84 (55,95)	3 (1,5)	91 (57,95)	5 (4,5)	77.5 (46,94)	4 (3,5)	85 (49,95)	16 (10,20)	67 (42,80)
Adaptive LASSO	4 (1,5)	80 (48,95)	3 (1,5)	83 (50,95)	5 (4,5)	81 (52,95)	4 (2,5)	82 (50,95)	11 (6,16)	63 (42,80)
Elastic Net	5 (1,5)	80 (0,95)	3 (1,5)	87 (0,95)	5 (4,5)	74 (26,95)	4 (3,5)	79 (13,95)	17 (10,20)	55 (0,78)
Iterated LASSO	4 (1,5)	86 (60,95)	3 (1,5)	91 (64,95)	5 (4,5)	84 (64,95)	4 (2,5)	88 (59,95)	12 (6,17)	75 (59,80)
Bootstrap-Enhanced LASSO-75	4 (2,5)	85 (64,94)	2 (1,5)	87 (72,95)	5 (4,5)	87 (76,95)	4 (2,5)	89 (79,95)	12 (7,17)	76 (70,80)
Weighted Fusion	5 (1,5)	62 (0,95)	3 (1,5)	94 (0,95)	5 (4,5)	74 (13,88)	4 (3,5)	77 (30,95)	20 (10,20)	51 (0,80)

Table 5 shows median and range of AUC results. Discriminatory predictive performance increased as the association with the outcome increased and also with larger sample size. In general, when considering scenarios with either weak associations of biomarkers with the outcome (scenarios 1 and 2) or scenarios with highly correlated variables (scenarios 2 and 4), the LASSO method showed good predictive ability over the other methods. The LASSO, EN, and WF performed comparably and outperformed the other methods. The BL-75 generally outperformed the AL. When considering scenario 5 with a moderate number of true signals with moderate correlation, the BL-75 showed the best predictive ability for the overweight outcome. In most scenarios, the IL performed poorly.

**Table 5:** Median (minimum, maximum) estimated AUC from 1000 simulated data sets

	*N* = 1000					*N* = 500				
Scenario	1	2	3	4	5	1	2	3	4	5
Overweight										
LASSO	0.57 (0.47,0.63)	0.61 (0.54,0.67)	0.77 (0.73,0.82)	0.79 (0.73,0.83)	0.77 (0.72,0.82)	0.54 (0.43,0.65)	0.60 (0.48,0.68)	0.77 (0.69,0.83)	0.78 (0.71,0.85)	0.77 (0.68,0.83)
Adaptive LASSO	0.55 (0.47,0.62)	0.58 (0.50,0.66)	0.72 (0.60,0.81)	0.73 (0.57,0.82)	0.76 (0.70,0.82)	0.54 (0.43,0.65)	0.57 (0.47,0.66)	0.72 (0.57,0.82)	0.73 (0.55,0.84)	0.75 (0.66,0.82)
Elastic Net	0.56 (0.47,0.63)	0.60 (0.54,0.67)	0.77 (0.73,0.82)	0.79 (0.73,0.83)	0.77 (0.73,0.82)	0.55 (0.43,0.65)	0.59 (0.49,0.68)	0.76 (0.69,0.83)	0.78 (0.71,0.85)	0.77 (0.68,0.83)
Iterated LASSO	0.52 (0.45,0.63)	0.54 (0.45,0.67)	0.54 (0.46,0.79)	0.57 (0.47,0.83)	0.70 (0.51,0.81)	0.52 (0.40,0.66)	0.54 (0.43,0.67)	0.55 (0.44,0.77)	0.58 (0.45,0.85)	0.70 (0.49,0.82)
Bootstrap-Enhanced LASSO-75	0.55 (0.48,0.62)	0.58 (0.48,0.65)	0.76 (0.71,0.82)	0.77 (0.71,0.82)	0.84 (0.79,0.88)	0.53 (0.42,0.65)	0.56 (0.44,0.66)	0.74 (0.64,0.82)	0.75 (0.65,0.83)	0.82 (0.73,0.88)
Weighted Fusion	0.56 (0.48,0.62)	0.60 (0.54,0.67)	0.77 (0.72,0.82)	0.78 (0.74,0.83)	0.78 (0.73,0.82)	0.55 (0.44,0.65)	0.59 (0.47,0.68)	0.77 (0.67,0.84)	0.78 (0.71,0.85)	0.77 (0.68,0.84)
Obese										
LASSO	0.78 (0.71,0.86)	0.80 (0.71,0.86)	0.94 (0.90,0.96)	0.96 (0.94,0.98)	0.99 (0.98,1.00)	0.76 (0.63,0.86)	0.79 (0.67,0.87)	0.93 (0.85,0.97)	0.96 (0.90,0.99)	0.98 (0.94,0.99)
Adaptive LASSO	0.73 (0.59,0.84)	0.74 (0.58,0.86)	0.88 (0.68,0.96)	0.92 (0.72,0.98)	0.98 (0.92,0.99)	0.72 (0.55,0.84)	0.73 (0.49,0.87)	0.89 (0.66,0.97)	0.93 (0.57,0.98)	0.96 (0.89,0.99)
Elastic Net	0.78 (0.71,0.86)	0.80 (0.71,0.86)	0.94 (0.90, 0.96)	0.96 (0.93,0.98)	0.99 (0.97,1.00)	0.76 (0.60,0.86)	0.79 (0.67,0.87)	0.93 (0.85,0.97)	0.96 (0.89,0.99)	0.98 (0.94,0.99)
Iterated LASSO	0.55 (0.45,0.79)	0.58 (0.43,0.85)	0.63 (0.46,0.96)	0.65 (0.33,0.98)	0.98 (0.76,1.00)	0.57 (0.42,0.80)	0.59 (0.42,0.87)	0.68 (0.44,0.95)	0.71 (0.34,0.98)	0.96 (0.64,0.99)
Bootstrap-Enhanced LASSO-75	0.76 (0.68, 0.82)	0.77 (0.68,0.85)	0.93 (0.88,0.97)	0.95 (0.91,0.98)	0.99 (0.82,1.00)	0.73 (0.57,0.85)	0.75 (0.62,0.85)	0.92 (0.83,0.97)	0.95 (0.88,0.98)	0.97 (0.79,0.99)
Weighted Fusion	0.78 (0.70,0.86)	0.80 (0.70,0.86)	0.94 (0.90, 0.96)	0.96 (0.64,0.98)	0.99 (0.97,1.00)	0.74 (0.60,0.86)	0.79 (0.66,0.87)	0.93 (0.86,0.97)	0.96 (0.88,0.98)	0.98 (0.96,1.00)

### Application results

A total of 86 biomarkers were considered for the application study. We identified the biomarkers that were consistently chosen across the six LASSO and LASSO-type methods and had a significant univariable association with the outcome. For the overweight outcome these were Adiponectin, Apolipoprotein H (ApoH), Calcitonin, soluble CD14 (sCD14), Complement 3 (C3), CRP, Ferritin, Growth Hormone (GH), Immunoglobulin M (IgM), Leptin, Myoglobin, Sex Hormone Binding Globulin (SHBG), and SPD and for the obese outcome were CRP, Interleukin-18 (IL-18), Leptin, Monocyte Chemotactic Protein-1 (MCP-1), SHBG, SPD, von Willebrand Factor (vWF), and YKL-40 (Table 6).

**Table 6:** TESAOD analysis, coefficients refer to associations of standardized biomarker values with overweight and obese separately

	Overweight							Obese						
	(*N* = 463 normal-weight, *N* = 306 overweight)							(*N* = 463 normal-weight, *N* = 66 obese)						
	LASSO	Adaptive LASSO	Elastic Net	Iterated LASSO	Bootstrap-Enhanced LASSO-75	Weighted Fusion	*p*^	LASSO	Adaptive LASSO	Elastic Net	Iterated LASSO	Bootstrap-Enhanced LASSO-75	Weighted Fusion	*p*^
Adiponectin	−0.2511	−0.3500	−0.2498	−0.3297	−0.3226	−0.2440	**	−0.0101	0	−0.0006	0	0	−0.0853	*
Apolipoprotein H	0.0571	0.0838	0.0762	0.1479	0.1309	0.0512	**	0.0881	0.1642	0.0346	0	0.2160	0.1277	**
Calcitonin^a^	0.0469	0.0765	0.0668	0.1681	0.0895	0.0352	*	0	0	0	0	0	0.0624	
Soluble CD14	−0.1133	−0.1679	−0.1296	−0.2492	−0.1923	−0.0994	*	−0.1020	−0.1706	−0.0488	0	−0.2272	−0.0965	
Complement 3	0.0847	0.1238	0.1183	0.2080	0.1483	0.0767	**	0.0899	0	0.0761	0	0	0.1306	**
C-Reactive Protein	0.2105	0.3150	0.2188	0.3791	0.2901	0.1923	**	0.5213	0.7636	0.4765	0.6802	0.6052	0.2526	**
Ferritin	0.1131	0.0969	0.1120	0.1431	0.1055	0.1115	**	0.0753	0	0.0469	0	0	0.0671	*
Growth Hormone	−0.1163	−0.1238	−0.1259	−0.1737	−0.1566	−0.1144	**	0	0	0	0	0	−0.0888	*
Immunoglobulin M	−0.0410	−0.0288	−0.0569	−0.1228	−0.0949	−0.0320	*	−0.1014	0	−0.0450	0	0	−0.1120	
Interleukin-18	0.0073	0.0047	0.0348	0.1515	0	0	**	0.1214	0.0653	0.0804	0.0774	0.2373	0.0870	*
Leptin	0.2057	0.3263	0.2102	0.3690	0.3177	0.1829	**	0.9186	1.1286	0.8548	1.2502	1.1551	0.3792	**
Monocyte Chemotactic Protein-1	0	0	0	0	0	0		0.1628	0.1471	0.1269	0.1792	0.2546	0.1275	**
Myoglobin	0.2323	0.3468	0.2300	0.3341	0.2856	0.2162	**	0.0391	0	0.0316	0	0	0.0829	*
Sex Hormone Binding Globulin	−0.2130	−0.2033	−0.1979	−0.2220	−0.2046	−0.2009	**	−0.4768	−0.6470	−0.4499	−0.7274	−0.4767	−0.2334	**
Surfactant Protein D	−0.1053	−0.1518	−0.1250	−0.2361	−0.2089	−0.0924	*	−0.3136	−0.4086	−0.2351	−0.3848	−0.4663	−0.2820	**
von Willebrand Factor	0	0	0	0	0	0		0.1980	0.0683	0.1737	0.2571	0.2694	0.1364	**
YKL-40	0	0	0.0108	0	0	0		0.1095	0.0847	0.0733	0.0314	0.1979	0.1151	**

^a^Categorized at median; ^: *p*-value for univariate association from binary logistic regression; *: *p* < 0.05; **: *p* < 0.01

The combination of both outcomes was also considered and 16 biomarkers were identified, namely Adiponectin, ApoH, Calcitonin, sCD14, C3, CRP, Ferritin, GH, IgM, IL-18, Leptin, MCP-1, Myoglobin, SHBG, SPD, and YKL-40 (Table 7).

**Table 7:** TESAOD analysis, coefficients refer to associations of standardized biomarker values with overweight and obese combined

	Overweight and Obese						
	(*N* = 463 normal-weight, *N* = 372 overweight and obese)						
	Lasso	Adaptive LASSO	Elastic Net	Iterated LASSO	Bootstrap-Enhanced LASSO-75	Weighted Fusion	*p*^
Adiponectin	−0.2143	−0.2770	−0.2210	−0.2827	−0.2334	−0.1985	**
Apolipoprotein H	0.0759	0.1047	0.0965	0.1028	0.1201	0.0628	**
Calcitonin^a^	0.0461	0.0647	0.0657	0.0782	0.1076	0.0236	*
Soluble CD14	−0.1439	−0.2052	−0.1598	−0.2411	−0.1681	−0.1143	*
Complement 3	0.1094	0.1174	0.1313	0.1307	0.1337	0.0923	**
C-Reactive Protein	0.2968	0.4064	0.3015	0.4235	0.3485	0.2630	**
Ferritin	0.1036	0.0834	0.1068	0.1397	0.0701	0.0988	**
Growth Hormone	−0.1155	−0.0898	−0.1181	−0.1430	−0.1529	−0.1158	**
Immunoglobulin M	−0.0883	−0.1068	−0.0940	−0.1568	−0.0982	−0.0715	*
Interleukin-18	0.0404	0.0379	0.0588	0.0677	0.0798	0.0223	**
Leptin	0.3312	0.4337	0.3499	0.4554	0.4052	0.2861	**
Monocyte Chemotactic Protein-1	0.0283	0.0829	0.0451	0.0572	0.0927	0.0030	*
Myoglobin	0.2267	0.3195	0.2286	0.2983	0.2587	0.2115	**
Sex Hormone Binding Globulin	−0.2827	−0.3609	−0.2739	−0.3612	−0.2703	−0.2593	**
Surfactant Protein D	−0.1612	−0.2086	−0.1794	−0.2439	−0.2401	−0.1328	**
YKL-40	0.0284	0.0278	0.0434	0.0222	0.0654	0.0152	*

^a^Categorized at median ^: *p*-value for univariate association from binary logistic regression; *: *p* < 0.05; **: *p* < 0.01

Results for all biomarkers, not limited to those chosen across all 6 LASSO and LASSO-type methods, can be found in Tables 11–13 in the Appendix.

## Discussion

In the simulation study, we compared the variable selection properties of the LASSO and five LASSO-type methods for the binary logistic regression model and did find certain situations in which one method outperformed the others. In general, when we considered scenarios with weak associations of biomarkers with the outcome and scenarios with high correlation between biomarkers, the IL tended to provide the most sparse solution, but had poor discriminatory predictive performance. The WF tended to correctly identify more of the true signals, but also incorrectly included more noise variables. Still, we were not able to identify one method that had an overall superior performance over the others. In general, our simulation set-up considered much smaller effects compared to those studied in the original methodological papers that proposed the LASSO [1], AL [3], EN [4], IL [6], BL [7], and WF [11] methods. This more realistic setting for biomarker research may contribute to why we did not see clear improvements of one method over the other.

Similar to our results, when comparing AL results to the LASSO, and considering both large and small effect sizes, Zou found that there was not one single method that consistently outperformed the others [3]. They found that in low sample size scenarios the LASSO performed the best with a low signal to noise ratio (SNR), while the AL outperformed the LASSO when high SNR was present [3]. In general, the LASSO was able to identify more of the non-zero coefficients as compared to the AL. In identifying the correct zero coefficients, both methods performed comparably with the exception of scenario 5 in which the LASSO outperformed the AL. Both the LASSO and AL methods have been shown to have good prediction accuracy [3], however the LASSO did show better discrimination than the AL in our simulation study. The EN has been shown to outperform the LASSO when collinearity is present. However, the EN typically chooses more variables than the LASSO, creating a less sparse solution [4]. For scenarios with high correlation (scenarios 2, 4, and 5), our results show that the EN correctly identified the true signals either comparably or better than the LASSO (Tables 3 and 4). As expected, the EN included more noise variables for all scenarios. Similarly, in our simulation study the LASSO provided a more sparse solution as compared to the EN (Table 2). The IL has been shown to provide a more sparse solution than the LASSO [6]. We confirmed in our simulation study that the IL tended to provide the most sparse solution as compared to the others, but it also demonstrated poor predictive ability. With sparse data-generating models, the BL has been shown to outperform the LASSO [8]. However, the BL has also been shown to be too stringent [8]. In the present study, in order to minimize the exclusion of any potentially important biomarkers, we a priori considered the BL-75. We found that when considering a moderate number of true signals with moderate correlation, the BL-75 showed the best predictive ability. WF has been shown to outperform the LASSO and EN [11]. Similar to the EN, WF also tends to over select variables and thus creates a less sparse solution. In our simulation study, we found that the WF correctly identified more true signals, but incorrectly included more noise variables.

Given these biomarker data, choice of optimal LASSO-type method was dependent on the characteristics of how the data were generated and should be guided by the research objectives. For objectives that aim to identify the maximal number of true signals, the WF was most optimal and the IL and AL the least. While identifying the greatest number of true signals, the WF also included more noise variables. The LASSO and EN performed well in the identification of many true signals and exclusion of more noise variables. For objectives that aim to maximize prediction, the LASSO, EN, and WF would also be optimal. While we found that no method had a clear overall advantage over the others, the IL was outperformed by the other methods in both variable selection and prediction. Additionally, while the BL showed the best predictive ability when considering a moderate number of true signals with moderate correlation, it was outperformed in variable selection. Given that the original LASSO method was not outperformed by the other methods, this method would be the most ideal method since it is the most direct and efficient method to implement. However, in general we recommend that the choice of optimal LASSO-type method should be guided by the underlying scientific question and by the research objectives.

When applying the methods to the TESAOD dataset, we considered three different outcomes, namely overweight, obese, and overweight or obese. We had expected similar biomarkers for both conditions and possibly stronger associations with obesity. We chose to combine overweight and obesity in a separate analysis as this would provide the highest power for most biomarkers as well as provide a summary measure. Considering overweight and obesity in separate models would allow us to estimate biomarker association levels specific to each condition. Not all biomarkers were chosen consistently across all methods when considering the overweight and obese outcomes separately. ApoH and sCD14 were consistently chosen across all methods for the overweight outcome and across all methods except for the IL for the obese outcome. Similarly, Adiponectin, C3, Ferritin, IgM and Myoglobin were consistently chosen across all methods for the overweight outcome and across all methods for the obese outcome except for AL, IL, and BL-75. Results from the simulation study suggest that the IL might not always choose the true signal and that the LASSO, EN, and WF might be more likely to identify the true signal as compared to AL and BL-75. Other biomarkers such as Calcitonin and GH were consistently chosen for the overweight outcome, but never chosen for the obese outcome. In contrast, MCP-1 and vWF were never chosen for the overweight outcome, but always chosen for the obese outcome. These differences may in part be due to the methodology and different sample sizes in the overweight and obese categories, but they also might be due to biological differences between the two outcomes.

Overall, in addition to the well-known effects of Adiponectin, CRP, and Leptin, the LASSO methods identified multiple biomarkers that have been reported to be associated with obesity and/or obesity related conditions [22–45] and that were largely classified into either a group of hormones or hormone related proteins (Adiponectin, Calcitonin, GH, Leptin, SHBG), or into a group of positive acute phase reactants and other biomarkers of inflammation (C3, CRP, Ferritin, IL-18, MCP-1, vWF, and YKL-40). The LASSO-type methods shrink coefficients and set other coefficients to 0, thus producing biased estimates. Of note, Tables 6 and 7 show biased estimates and the magnitude of the estimation differences could be noted. The tuning parameter chosen by cross validation affects the amount of shrinkage and this tuning parameter may differ between LASSO-type methods.

### Strengths and limitations

A strength of this study is that real-world data were used in the development of the simulation study parameters. In particular, the scenarios that were considered represent a more realistic setting that might be present in high dimensional serum biomarker research such as sparse number of true signals, weak to moderate association of the biomarkers with the outcomes, and high correlation between biomarkers. In the application study we confirmed potentially important biomarkers of overweight and obesity using results that were consistent across six LASSO and LASSO-type methods.

A limitation to this study is that although we found that in general the IL tended to provide the most sparse solution and the WF tended to correctly identify the most number of true signals, the choice of optimal LASSO method is data structure dependent and results from this study may not be generalizable to other biomarker studies. Furthermore, for the BL method, rather than consider a strict intersection, we considered the BL-75. We acknowledge that this may not fully optimize results and a different frequency threshold may outperform the BL-75. In addition, the primary goal of this research was to study the binary logistic regression model. Particularly, we were interested in how the LASSO-type models would compare when considering a common (40% overweight) or less common (12% obese) outcome. Considering both outcomes together as a single ordinal or as a multinomial response might have been more efficient than to consider them as separate binary responses and performance of LASSO-type approaches for such outcome measures will be a future research topic.

In this paper we focus on situations where the number of variables p is smaller than the sample size N. As shown in Fan and Fan [46], Fan and Liv [47], and Fan, Samworth, and Wu [48], when p > N, performance of the LASSO-type methods is inferior to that of certain two-stage methods, which first apply a screening procedure to reduce the number of important variables below the sample size, then apply methods like LASSO to the selected subset of variables. Comparison of such two-stage methods for the p > N situation is beyond the scope of this paper and will be the topic of future investigation.

## Conclusions

For the data scenarios examined, the LASSO method was overall not outperformed by any of the other methods. Choice of optimal LASSO-type method was dependent on characteristics of how the data were generated. In general, these characteristics are unknown and can only to some extent be estimated. Nevertheless, choice of optimal LASSO-type method should be guided by knowledge of the underlying scientific question and by the research objectives. The LASSO-type methods were able to identify biomarkers that were known to be associated with obesity and obesity related conditions, demonstrating the promise of such methods in future investigations.

## Appendix

**Table 8:** HumanMAP version 1.6 biomarkers

Adiponectin	Granulocyte-Macrophage Colony-Stimulating Factor	Macrophage-Derived Chemokine
Alpha-1-Antitrypsin	Growth Hormone	Macrophage Inflammatory Protein 1-alpha
Alpha-Fetoprotein	Haptoglobin	Macrophage Inflammatory Protein-1 beta
Alpha-2-Macroglobulin	Immunoglobulin A	Matrix Metalloproteinase-2
Apolipoprotein A-1	Immunoglobulin E	Matrix Metalloproteinase-3
Apolipoprotein C-III	Immunoglobulin M	Matrix Metalloproteinase-9
Apolipoprotein H	Insulin	Monocyte Chemotactic Protein 1
Beta-2 Microglobulin	Intercellular Adhesion Molecule-1	Myeloperoxidase
Brain Derived Neurotrophic Factor	Interferon-gamma	Myoglobin
Calcitonin	Interleukin-1 alpha	Plasminogen Activator Inhibitor 1
Cancer Antigen 19-9	Interleukin-1 beta	Pregnancy-Associated Plasma Protein a
Cancer Antigen 125	Interleukin-1 Receptor Antagonist	Prostate Specific Antigen, Free
Carcinoembryonic Antigen	Interleukin-2	Prostatic Acid Phosphatase
CD40	Interleukin-3	T-Cell Specific Protein, Regulated upon Activation Normal T-cell Expressed, and presumably Secreted
CD40 Ligand	Interleukin-4	Serum Amyloid P
Complement 3	Interleukin-5	Serum Glutamic Oxaloacetic Transminase
Creatine Kinase-MB	Interleukin-6	Sex Hormone Binding Globulin
Endothelin-1	Interleukin-7	Stem Cell Factor
Eotaxin	Interleukin-8	Thrombopoietin
Epidermal Growth Factor	Interleukin-10	Thyroxine Binding Globulin
Epithelial-Derived Neutrophil-Activating Protein-78	Interleukin-12 subunit p40	Thyroid Stimulating Hormone
Erythropoietin	Interleukin-12 subunit p70	Tissue Factor
Extracellular Newly Identified RAGE-binding protein	Interleukin-13	Tissue Inhibitor of Metalloproteinase 1
Factor VII	Interleukin-15	Tumor Necrosis Factor-alpha
Fatty Acid Binding Protein	Interleukin-16	Tumor Necrosis Factor-beta
Ferritin	Interleukin-18	Tumor Necrosis Factor RII
Fibrinogen	Leptin	Vascular Cell Adhesion Molecule-1
Fibroblast Growth Factor-Basic	Lipoprotein (a)	Vascular Endothelial Growth Factor
Granulocyte Colony-Stimulating Factor	Lymphotactin	von Willebrand Factor

**Table 9:** Biomarkers measured at the Arizona Respiratory Center

Soluble CD14	
Club (Clara) Cell Secretory Protein	
C-Reactive Protein	
Surfactant Protein D	
YKL-40

**Table 10:** Measurements of biomarker concentrations

Category description	Reported from lab	Decision on how to use data
Undetectable values	Reflect samples below the lowest standard	The lowest observed value for each biomarker was identified and all low values for that biomarker were recorded as ½ that value (Myriad-RBM) or ½ the lowest standard (ARC)
Normal values	Normal values	Normal Values
High values	Reflect samples above the highest standard	Value equals twice the highest value (Myriad-RBM) or twice the highest standard (ARC)

**Table 11:** TESAOD application, overweight subjects (*N* = 463 normal-weight, *N* = 306 overweight)

	Lasso	Adaptive LASSO	Elastic Net	Iterated LASSO	Bootstrap-Enhanced LASSO-75	Weighted Fusion	Logistic Regression, Coefficient (*p*-value)
Adiponectin	−0.2511	−0.3500	−0.2498	−0.3297	−0.3226	−0.2440	−0.5188 (<0.001)
Alpha-1 Antitrypsin	−0.0283	−0.0283	−0.0554	−0.1381	0	−0.0210	−0.1552 (0.038)
Alpha-Fetoprotein	0	0	0	0	0	0	0.1012 (0.178)
Alpha-2 Macroglobulin	−0.0005	0	−0.0214	0	0	−0.0014	−0.3183 (<0.001)
Apolipoprotein A-1	0	0	0	0	0	0	−0.0502 (0.497)
Apolipoprotein C-III	0	0	0	0	0	0	0.2049 (0.006)
Apolipoprotein H	0.0571	0.0838	0.0762	0.1479	0.1309	0.0512	0.3348 (<0.001)
Beta-2 Microglobulin	0	0	0	0	0	0	0.1495 (0.044)
Brain Derived Neurotrophic Factor	0	0	0	0	0	0	0.0412 (0.576)
Calcitonin^a^	0.0469	0.0765	0.0668	0.1681	0.0895	0.0352	0.3435 (0.020)
Cancer Antigen 19-9	0	0	0	0	0	0	−0.0007 (0.992)
Cancer Antigen 125	0	0	0	0	0	0	0.0724 (0.329)
Carcinoembryonic Antigen	0	0	0	0	0	0	0.0157 (0.831)
Soluble CD14	−0.1133	−0.1679	−0.1296	−0.2492	−0.1923	−0.0994	−0.1716 (0.023)
CD40	0	0	0	0	0	0	0.0302 (0.682)
CD40Ligand	0	0	0	0	0	0	−0.0096 (0.896)
Club Cell Secretory Protein	0	0	0	0	0	0	0.0107 (0.885)
Complement 3	0.0847	0.1238	0.1183	0.2080	0.1483	0.0767	0.3855 (<0.001)
C-Reactive Protein	0.2105	0.3150	0.2188	0.3791	0.2902	0.1923	0.3537 (<0.001)
Creatine Kinase-MB	0	0	0.0062	0	0	0	0.2130 (0.005)
Endothelin-1^b^	0	0	0	0	0	0	−0.0262 (0.874)
Eotaxin	−0.0451	−0.0904	−0.0693	−0.1443	−0.1277	−0.0335	−0.0946 (0.206)
Epidermal Growth Factor	−0.0140	−0.0591	−0.0396	−0.1180	−0.0689	−0.0041	−0.1000 (0.174)
Epithelial-Derived Neutrophil-Activating Protein-78	0	0	0	0	0	0	−0.0065 (0.930)
Erythropoietin^a^	0	0	0	0	0	0	0.0914 (0.535)
Extracellular Newly Identified RAGE-Binding Protein	0	0	0	0	0	0	0.0347 (0.637)
Fatty Acid Binding Protein	0	0	0	0	0	0	0.2592 (0.001)
Ferritin	0.1131	0.0969	0.1120	0.1431	0.1055	0.1115	0.4215 (<0.001)
Fibrinogen	0	0	0	0	0	0	0.1263 (0.087)
Fibroblast Growth Factor-Basic^b^	0	0	0	0	0	0	0.1792 (0.251)
Granulocyte Colony-Stimulating Factor	−0.0713	−0.1647	−0.0879	−0.2319	−0.1709	−0.0547	−0.1155 (0.117)
Granulocyte-Macrophage Colony-Stimulating Factor^b^	0.0255	0	0.0445	0.1224	0	0.0153	0.4067 (0.036)
Growth Hormone	−0.1163	−0.1238	−0.1259	−0.1737	−0.1566	−0.1144	−0.4159 (<0.001)
Haptoglobin	0	0	0	0	0	0	0.1710 (0.023)
Immunoglobulin A	0	0	0	0	0	0	0.1227 (0.094)
Immunoglobulin E	0	0	0	0	0	0	0.1331 (0.074)
Immunoglobulin M	−0.0410	−0.0288	−0.0569	−0.1228	−0.0949	−0.0320	−0.1509 (0.041)
Intercellular Adhesion Molecule-1	0	0	0	0	0	0	0.0608 (0.411)
Interferon-Gamma^a^	0	0	0	0	0	0	−0.0739 (0.616)
Interleukin-1alpha^b^	0	0	−0.0097	0	0	0	−0.2350 (0.134)
Interleukin-1beta^a^	0	0	0	0	0	0	0.1519 (0.303)
Interleukin-1 Receptor Antagonist	0	0	0	0	0	0	0.0141 (0.849)
Interleukin-3^b^	0	0	0.0027	0	0.0167	0	0.0991 (0.504)
Interleukin-4^b^	0	0	−0.0022	0	0	0	−0.2057 (0.172)
Interleukin-5^a^	0	0	0	0	0	0	−0.0003 (0.998)
Interleukin-6^a^	0	0	0	0	0	0	0.1995 (0.176)
Interleukin-7^b^	0	0	0.0014	0	0	0	0.1563 (0.292)
Interleukin-8	0	0.0155	0.0089	0	0	0	0.0810 (0.269)
Interleukin-10^b^	0	0	0	0	0	0	0.1657 (0.316)
Interleukin-12p40^b^	−0.0089	0	−0.0351	−0.1216	0	0	−0.2502 (0.146)
Interleukin-13	0	0	0	0	0	0	−0.0110 (0.881)
Interleukin-15^a^	0	0	0	0	0	0	−0.1304 (0.376)
Interleukin-16	0	−0.0597	0	0	0	0	0.0584 (0.433)
Interleukin-18	0.0073	0.0047	0.0348	0.1515	0	0	0.2131 (0.005)
Leptin	0.2057	0.3263	0.2102	0.3690	0.3177	0.1829	0.2062 (0.008)
Lipoprotein (a)	0	0	0	0	0	0	−0.1247 (0.094)
Macrophage-Derived Chemokine	0	0	0	0	0	0	0.0477 (0.517)
Macrophage Inflammatory Protein-1alpha	0	0	0	0	0	0	0.1057 (0.156)
Macrophage Inflammatory Protein-1beta	0.0002	0	0.0307	0	0	0	0.1679 (0.026)
Matrix Metalloproteinase-2^a^	0	0	0	0	0	0	0.0212 (0.886)
Matrix Metalloproteinase-3	0	0	0	0	0	0	0.1937 (0.010)
Matrix Metalloproteinase-9^a^	0	0	0	0	0	0	0.1040 (0.480)
Monocyte Chemotactic Protein-1	0	0	0	0	0	0	0.1189 (0.110)
Myeloperoxidase	−0.0222	0	−0.0378	−0.1046	0	−0.0113	−0.0582 (0.430)
Myoglobin	0.2323	0.3468	0.2300	0.3341	0.2856	0.2162	0.4412 (<0.001)
Plasminogen Activator Inhibitor 1	0	0	0	0	0	0	0.1848 (0.014)
Pregnancy-Associated Plasma Protein a	0	0	0	0	0	0	−0.0047 (0.949)
Prostatic Acid Phosphatase^a^	0	0	0	0	0	0	0.2134 (0.148)
T-Cell Specific Protein RANTES	0	0	0	0	0	0	0.0804 (0.278)
Serum Amyloid P	0.1352	0	0.1196	0.0851	0.1133	0.1346	0.5442 (<0.001)
Serum Glutamic Oxaloacetic Transminase	0	0	0.0206	0	0	0	0.0672 (0.364)
Sex Hormone Binding Globulin	−0.2130	−0.2033	−0.1979	−0.2220	−0.2046	−0.2009	−0.5329 (<0.001)
Stem Cell Factor	0	0	0	0	0	0	0.0395 (0.591)
Surfactant Protein D	−0.1053	−0.1518	−0.1250	−0.2361	−0.2089	−0.0924	−0.1774 (0.020)
Thrombopoietin	0	0	0	0	0	0	0.0218 (0.767)
Thyroid Stimulating Hormone	0	0	−0.0080	0	0	0	−0.0191 (0.795)
Thyroxine Binding Globulin	0	0	0	0	0	0	−0.1083 (0.150)
Tissue Factor^b^	0	0	0	0	0	0	−0.0137 (0.945)
Tissue Inhibitor of Metalloproteinase 1	0	0	0	0	0	0	0.1395 (0.059)
Tumor Necrosis Factor-alpha	0	0	0	0	0	0	0.0616 (0.407)
Tumor Necrosis Factor-beta^b^	0	0	0	0	0	0	−0.0666 (0.682)
Tumor Necrosis Factor RII	0	0	0	0	0	0	0.1413 (0.056)
Vascular Cell Adhesion Molecule-1	0	0	0	0	0	0	0.0698 (0.344)
Vascular Endothelial Growth Factor	0	0	0	0	0	0	0.1098 (0.138)
von Willebrand Factor	0	0	0	0	0	0	0.0579 (0.430)
YKL-40	0	0	0.0108	0	0	0	0.1184 (0.108)

^a^Categorized at median; ^b^Categorized at detection limit

**Table 12:** TESAOD application, obese subjects (*N* = 463 normal-weight, *N* = 66 obese)

	Lasso	Adaptive LASSO	Elastic Net	Iterated LASSO	Bootstrap-Enhanced LASSO-75	Weighted Fusion	Logistic Regression, Coefficient (*p*-value)
Adiponectin	−0.0101	0	−0.0006	0	0	−0.0853	−0.3089 (0.018)
Alpha-1 Antitrypsin	0	0	0	0	0	−0.0320	−0.0748 (0.573)
Alpha-Fetoprotein	−0.0256	0	0	0	0	−0.0730	0.0009 (0.994)
Alpha-2 Macroglobulin	−0.0187	0	−0.0065	0	0	−0.1110	−0.3163 (0.022)
Apolipoprotein A-1	−0.2167	−0.3546	−0.1691	−0.3127	−0.2627	−0.1011	−0.1488 (0.260)
Apolipoprotein C-III	0	0	0	0	0	0	0.1865 (0.121)
Apolipoprotein H	0.0881	0.1642	0.0346	0	0.2160	0.1277	0.5315 (<0.001)
Beta-2 Microglobulin	0	0	0	0	0	0.0417	0.3586 (0.005)
Brain Derived Neurotrophic Factor	0	0	0	0	0	0	0.2784 (0.024)
Calcitonin^a^	0	0	0	0	0	0.0624	0.2469 (0.349)
Cancer Antigen 19-9	−0.1216	−0.1226	−0.0633	0	−0.3059	−0.1439	−0.1357 (0.269)
Cancer Antigen 125	0	0	0	0	0	−0.0081	0.0942 (0.484)
Carcinoembryonic Antigen	−0.0516	0	0	0	−0.1779	−0.1107	−0.1338 (0.301)
Soluble CD14	−0.1020	−0.1706	−0.0488	0	−0.2272	−0.0965	−0.0801 (0.544)
CD40	0	0	0	0	0	−0.0167	0.2122 (0.109)
CD40Ligand	−0.0176	0	0	0	0	−0.0808	−0.0731 (0.580)
Club Cell Secretory Protein	0	0	0	0	0	−0.0705	−0.1442 (0.263)
Complement 3	0.0899	0	0.0761	0	0	0.1306	0.5991 (<0.001)
C-Reactive Protein	0.5213	0.7636	0.4765	0.6802	0.6052	0.2526	0.7864 (<0.001)
Creatine Kinase-MB	0.0818	0.1652	0.0423	0	0.1442	0.0957	0.2967 (0.045)
Endothelin-1^b^	0	0	0	0	0	0.0487	0.2000 (0.481)
Eotaxin	−0.0965	−0.1557	−0.0581	0	−0.2893	−0.1414	−0.2317 (0.111)
Epidermal Growth Factor	0	0	0	0	0	0.0190	0.1794 (0.198)
Epithelial-Derived Neutrophil-Activating Protein-78	0	0	0	0	0	0.0353	0.2525 (0.024)
Erythropoietin^a^	0	0	0	0	0	−0.0174	0.0736 (0.780)
Extracellular Newly Identified RAGE-Binding Protein	0	0	0	0	0	0.0092	0.0324 (0.803)
Fatty Acid Binding Protein	0	0	0	0	0	0	0.3317 (0.029)
Ferritin	0.0753	0	0.0469	0	0	0.0671	0.2900 (0.038)
Fibrinogen	0	0	0	0	0	0.0168	0.1290 (0.320)
Fibroblast Growth Factor-Basic^b^	0	0	0	0	0	0.0518	0.4799 (0.074)
Granulocyte Colony-Stimulating Factor	0	0	0	0	0	0.0705	0.3130 (0.027)
Granulocyte-Macrophage Colony-Stimulating Factor^b^	0	0	0	0	0	0.0086	0.2726 (0.430)
Growth Hormone	0	0	0	0	0	−0.0888	−0.2576 (0.049)
Haptoglobin	0	0	0	0	0	0.0107	0.2537 (0.027)
Immunoglobulin A	0	0	0	0	0	−0.0046	0.1143 (0.358)
Immunoglobulin E	0	0	0	0	0	0	−0.0469 (0.720)
Immunoglobulin M	−0.1014	0	−0.0450	0	0	−0.1120	−0.1411 (0.278)
Intercellular Adhesion Molecule-1	0	0	0	0	0	0	0.1826 (0.167)
Interferon-Gamma^a^	0.0820	0.0300	0.0245	0	0.1199	0.1218	0.3461 (0.195)
Interleukin-1alpha^b^	0	0	0	0	0	0.0154	0.1322 (0.624)
Interleukin-1beta^a^	0	0	0	0	0	0.0535	0.2558 (0.333)
Interleukin-1 Receptor Antagonist	0	−0.0844	0	0	0	−0.0275	0.1284 (0.353)
Interleukin-3^b^	0	0	0	0	0	−0.0185	0.1956 (0.458)
Interleukin-4^b^	0	0	0	0	0	0	−0.0315 (0.906)
Interleukin-5^a^	0	0	0	0	0	−0.0203	−0.0217 (0.934)
Interleukin-6^a^	0	0	0	0	0	−0.0069	0.4395 (0.098)
Interleukin-7^b^	0.1132	0	0.0761	0.012	0	0.1345	0.6057 (0.023)
Interleukin-8	−0.0407	0	0	0	0	−0.0765	−0.0588 (0.665)
Interleukin-10^b^	0	0	0	0	0	0.0124	0.4472 (0.110)
Interleukin-12p40^b^	0	0	0	0	0	0.0103	0.2217 (0.435)
Interleukin-13	0	0	0	0	0	−0.0653	−0.0841 (0.533)
Interleukin-15^a^	0	0	0	0	0	−0.0037	0.1434 (0.587)
Interleukin-16	0	0	0	0	0	0.0531	0.1822 (0.175)
Interleukin-18	0.1214	0.0653	0.0804	0.0774	0.2373	0.0870	0.3215 (0.015)
Leptin	0.9186	1.1286	0.8548	1.2502	1.1505	0.3792	1.3928 (<0.001)
Lipoprotein (a)	0	0	0	0	0	0	−0.0010 (0.994)
Macrophage-Derived Chemokine	0	0	0	0	0	0.0476	0.2533 (0.042)
Macrophage Inflammatory Protein-1alpha	0	0	0	0	0	0.0531	0.2623 (0.057)
Macrophage Inflammatory Protein-1beta	0	0	0	0	0	0.0241	0.2934 (0.022)
Matrix Metalloproteinase-2^a^	0.1090	0	0.0776	0.0443	0.1443	0.1242	0.6992 (0.011)
Matrix Metalloproteinase-3	0	0	0	0	0	−0.0308	−0.0218 (0.868)
Matrix Metalloproteinase-9^a^	0.0101	0.0832	0	0	0	0.0606	0.3084 (0.244)
Monocyte Chemotactic Protein-1	0.1628	0.1471	0.1269	0.1792	0.2546	0.1275	0.4521 (0.001)
Myeloperoxidase	0	−0.0542	0	0	0	−0.0927	−0.1054 (0.425)
Myoglobin	0.0391	0	0.0316	0	0	0.0829	0.3239 (0.011)
Plasminogen Activator Inhibitor 1	0	0	0	0	0	0.0454	0.4603 (0.001)
Pregnancy-Associated Plasma Protein a	0	0	0	0	0	−0.0995	−0.1306 (0.306)
Prostatic Acid Phosphatase^a^	0	0	0	0	0	−0.0477	−0.0652 (0.805)
T-Cell Specific Protein RANTES	0	0	0	0	0	0.0444	0.4370 (0.002)
Serum Amyloid P	0	0	0.0019	0	0	0.0836	0.6526 (<0.001)
Serum Glutamic Oxaloacetic Transminase	0.0340	0.0234	0	0	0	0.0851	0.0350 (0.791)
Sex Hormone Binding Globulin	−0.4768	−0.6470	−0.4499	−0.7274	−0.4767	−0.2334	−0.5552 (<0.001)
Stem Cell Factor	0	0	0	0	0	0.0212	0.3146 (0.008)
Surfactant Protein D	−0.3136	−0.4086	−0.2351	−0.3848	−0.4663	−0.2820	−0.4833 (0.002)
Thrombopoietin	0.0243	0	0.0147	0	0	0.0933	0.3849 (0.002)
Thyroid Stimulating Hormone	−0.0264	−0.0034	0	0	0	−0.0515	−0.1010 (0.424)
Thyroxine Binding Globulin	0	0	0	0	0	0	0.0722 (0.570)
Tissue Factor^b^	0	0	0	0	0	0.0788	0.6627 (0.029)
Tissue Inhibitor of Metalloproteinase 1	0	0	0	0	0	−0.0023	0.2617 (0.031)
Tumor Necrosis Factor-alpha	0	0	0	0	0	0.0380	0.2958 (0.044)
Tumor Necrosis Factor-beta^b^	0.0165	0	0	0	0	0.0848	0.2309 (0.406)
Tumor Necrosis Factor RII	0	0	0	0	0	0.0287	0.3291 (0.011)
Vascular Cell Adhesion Molecule-1	0	0.1194	0	0	0	0.0828	0.2680 (0.043)
Vascular Endothelial Growth Factor	0	0	0	0	0	0	0.3250 (0.015)
von Willebrand Factor	0.1980	0.0683	0.1737	0.2571	0.2694	0.1364	0.4185 (<0.001)
YKL-40	0.1095	0.0847	0.0733	0.0314	0.1979	0.1151	0.3671 (0.005)

^a^Categorized at median; ^b^Categorized at detection limit

**Table 13:** TESAOD application, overweight and obese subjects combined (*N* = 463 normal-weight, *N* = 372 overweight and obese)

	Lasso	Adaptive LASSO	Elastic Net	Iterated LASSO	Bootstrap-Enhanced LASSO-75	Weighted Fusion	Logistic Regression, Coefficient (*p*-value)
Adiponectin	−0.2143	−0.2770	−0.2210	−0.2827	−0.2334	−0.1985	−0.4799 (<0.001)
Alpha-1 Antitrypsin	−0.0311	0	−0.0464	0	0	−0.0199	−0.1415 (0.045)
Alpha-Fetoprotein	0	0	0	0	0	0	0.0833 (0.238)
Alpha-2 Macroglobulin	−0.0435	0	−0.0580	−0.0276	−0.0705	−0.0348	−0.3158 (<0.001)
Apolipoprotein A-1	−0.0105	−0.0257	−0.0324	0	0	0	−0.0684 (0.328)
Apolipoprotein C-III	0	0	0	0	0	0	0.2077 (0.003)
Apolipoprotein H	0.0759	0.1047	0.0965	0.1028	0.1201	0.0628	0.3683 (<0.001)
Beta-2 Microglobulin	0	0	0	0	0	0	0.1925 (0.007)
Brain Derived Neurotrophic Factor	0	0	0	0	0	0	0.0886 (0.206)
Calcitonin^a^	0.0461	0.0647	0.0657	0.0782	0.1076	0.0236	0.3263 (0.020)
Cancer Antigen 19-9	0	0	0	0	0	0	−0.0265 (0.703)
Cancer Antigen 125	0	0	0	0	0	0	0.0769 (0.272)
Carcinoembryonic Antigen	0	0	0	0	0	0	−0.0118 (0.866)
Soluble CD14	−0.1439	−0.2052	−0.1598	−0.2411	−0.1681	−0.1143	−0.1542 (0.030)
CD40	0	−0.0075	−0.0085	0	0	0	0.0618 (0.376)
CD40Ligand	0	0	0	0	0	0	−0.0206 (0.768)
Club Cell Secretory Protein	0	0	−0.0009	0	0	0	−0.0177 (0.799)
Complement 3	0.1094	0.1174	0.1313	0.1307	0.1337	0.0923	0.4334 (<0.001)
C-Reactive Protein	0.2968	0.4064	0.3015	0.4235	0.3485	0.2630	0.4345 (<0.001)
Creatine Kinase-MB	0.0200	0	0.0344	0	0	0.0087	0.2235 (0.002)
Endothelin-1^b^	0	0	0	0	0	0	0.0155 (0.921)
Eotaxin	−0.1066	−0.1808	−0.1291	−0.2042	−0.1816	−0.0717	−0.1164 (0.099)
Epidermal Growth Factor	0	0	0	0	0	0	−0.0540 (0.438)
Epithelial-Derived Neutrophil-Activating Protein-78	0	0	0	0	0	0	0.0507 (0.466)
Erythropoietin^a^	0	0	0	0	0	0	0.0883 (0.526)
Extracellular Newly Identified RAGE-Binding Protein	0	0	0	0	0	0	0.0343 (0.622)
Fatty Acid Binding Protein	0	0	0	0	0	0	0.2676 (<0.001)
Ferritin	0.1036	0.0834	0.1068	0.1397	0.0701	0.0988	0.3946 (<0.001)
Fibrinogen	0	0	0	0	0	0	0.1273 (0.069)
Fibroblast Growth Factor-Basic^b^	0	0	0.0100	0	0	0	0.2341 (0.111)
Granulocyte Colony-Stimulating Factor	−0.0564	−0.1372	−0.0756	−0.1276	−0.1268	−0.0252	−0.0437 (0.530)
Granulocyte-Macrophage Colony-Stimulating Factor^b^	0.0175	0	0.0320	0	0.0604	0.0066	0.3837 (0.038)
Growth Hormone	−0.1155	−0.0898	−0.1181	−0.1430	−0.1529	−0.1158	−0.3918 (<0.001)
Haptoglobin	0	0	0	0	0	0	0.1949 (0.007)
Immunoglobulin A	0	0	0	0	0	0	0.1245 (0.075)
Immunoglobulin E	0	0	0	0	0	0	0.1028 (0.142)
Immunoglobulin M	−0.0883	−0.1068	−0.0940	−0.1568	−0.0982	−0.0715	−0.1486 (0.034)
Intercellular Adhesion Molecule-1	0	0	0	0	0	0	0.0842 (0.229)
Interferon-Gamma^a^	0	0	0	0	0	0	−0.0001 (0.999)
Interleukin-1alpha^b^	0	0	0	0	0	0	−0.1667 (0.257)
Interleukin-1beta^a^	0	0	0.0022	0	0	0	0.1703 (0.222)
Interleukin-1 Receptor Antagonist	−0.0026	0	−0.0219	0	0	0	0.0328 (0.638)
Interleukin-3^b^	0	0	0	0	0	0	0.1162 (0.406)
Interleukin-4^b^	0	0	0	0	0	0	−0.1744 (0.219)
Interleukin-5^a^	0	0	0	0	0	0	−0.0041 (0.976)
Interleukin-6^a^	0	0	0	0	0	0	0.2417 (0.083)
Interleukin-7^b^	0.0157	0	0.0348	0	0	0	0.2358 (0.092)
Interleukin-8	0	0	0	0	0	0	0.0582 (0.403)
Interleukin-10^b^	0	0	0	0	0	0	0.2179 (0.161)
Interleukin-12p40^b^	0	0	−0.0170	0	0	0	−0.1583 (0.322)
Interleukin-13	−0.0057	0	−0.0236	0	0	0	−0.0239 (0.732)
Interleukin-15^a^	0	0	0	0	0	0	−0.0819 (0.557)
Interleukin-16	0	−0.0243	0	0	0	0	0.0790 (0.263)
Interleukin-18	0.0404	0.0379	0.0588	0.0677	0.0798	0.0223	0.2362 (0.001)
Leptin	0.3312	0.4337	0.3499	0.4554	0.4052	0.2861	0.3234 (<0.001)
Lipoprotein (a)	0	0	0	0	0	0	−0.1016 (0.147)
Macrophage-Derived Chemokine	0	0	0	0	0	0	0.0860 (0.217)
Macrophage Inflammatory Protein-1alpha	0	0	0	0	0	0	0.1323 (0.061)
Macrophage Inflammatory Protein-1beta	0	0	0.0115	0	0	0	0.1924 (0.008)
Matrix Metalloproteinase -2^a^	0	0	0	0	0	0	0.1380 (0.322)
Matrix Metalloproteinase-3	0	0	0	0	0	0	0.1560 (0.027)
Matrix Metalloproteinase-9^a^	0	0	0	0	0	0	0.1401 (0.315)
Monocyte Chemotactic Protein-1	0.0283	0.0829	0.0451	0.0572	0.0927	0.0030	0.1757 (0.013)
Myeloperoxidase	−0.0543	−0.0728	−0.0713	−0.1149	−0.0469	−0.0239	−0.0670 (0.337)
Myoglobin	0.2267	0.3195	0.2286	0.2983	0.2587	0.2115	0.4299 (<0.001)
Plasminogen Activator Inhibitor 1	0	0	0	0	0	0	0.2314 (0.001)
Pregnancy-Associated Plasma Protein a	0	0	0	0	0	0	−0.0279 (0.689)
Prostatic Acid Phosphatase^a^	0	0	0	0	0	0	0.1638 (0.240)
T-Cell Specific Protein RANTES	0	0	0	0	0	0	0.1368 (0.052)
Serum Amyloid P	0.1028	0	0.0961	0.0668	0	0.1157	0.5657 (<0.001)
Serum Glutamic Oxaloacetic Transminase	0.0380	0	0.0561	0.0795	0	0.0122	0.0617 (0.378)
Sex Hormone Binding Globulin	−0.2827	−0.3609	−0.2739	−0.3612	−0.2703	−0.2593	−0.5321 (<0.001)
Stem Cell Factor	0	0	0	0	0	0	0.0931 (0.182)
Surfactant Protein D	−0.1612	−0.2086	−0.1794	−0.2439	−0.2401	−0.1328	−0.2215 (0.002)
Thrombopoietin	0	0	0	0	0	0	0.0924 (0.186)
Thyroid Stimulating Hormone	−0.0207	−0.0430	−0.0379	−0.0273	0	0	−0.0352 (0.613)
Thyroxine Binding Globulin	0	0	0	0	0	0	−0.0751 (0.285)
Tissue Factor^b^	0	0	0	0	0	0	0.1279 (0.489)
Tissue Inhibitor of Metalloproteinase 1	0	0	0	0	0	0	0.1665 (0.018)
Tumor Necrosis Factor-alpha	0	0	0	0	0	0	0.0982 (0.162)
Tumor Necrosis Factor-beta^b^	0	0	0	0	0	0	−0.0113 (0.941)
Tumor Necrosis Factor RII	0	0	0	0	0	0	0.1797 (0.011)
Vascular Cell Adhesion Molecule-1	0.0333	0.0473	0.0502	0.0654	0	0.0103	0.1073 (0.125)
Vascular Endothelial Growth Factor	0	0	0	0	0	0	0.1468 (0.036)
von Willebrand Factor	0	0	0.0039	0	0	0	0.1308 (0.061)
YKL-40	0.0284	0.0278	0.0434	0.0222	0.0654	0.0152	0.1619 (0.021)

^a^Categorized at median; ^b^Categorized at detection limit

## Abbreviations

AL: Adaptive LASSO
ApoH: Apolipoprotein H
ARC: Arizona respiratory center
AUC: Area under the receiver operating characteristic curve
BL: Bootstrap-Enhanced LASSO
C3: Complement 3
CC16: Club cell secretory protein16
CRP: C-Reactive Protein
EN: Elastic net
GH: Growth hormone
IgM: Immunoglobulin M
IL: Iterated LASSO
IL-18: Interleukin-18
LASSO: Least absolute shrinkage and selection operator
LDD: Least detectable dose
MCP-1: Monocyte chemotactic protein-1
RBM: Rules based medicine
sCD14: soluble CD14
SHBG: Sex hormone binding globulin
SPD: Surfactant protein D
TESAOD: Tucson epidemiological study of airway obstructive disease
vWF: von Willebrand Factor
WF: Weighted fusion
